# Supplementary material for: Profiles of Cytokinins Metabolic Genes and Endogenous Cytokinins Dynamics during Shoot Multiplication In Vitro of Phalaenopsis
Source: Int J Mol Sci. 2022 Mar 29;23(7):3755. doi: 10.3390/ijms23073755 (PMC8998587; doi:10.3390/ijms23073755)

Supplementary information

Table S1. List of primers used in this study.

Gene name	Forward primer (5-3')	Reverse primer (5-3')
PaIPT1	GGTAGTGAACGCCGACAAGA	CAGCGAGAGATCGAAACCCC
PaIPT2	CTCGAGGACTGAAGCAAGCA	TTCCGTTCAAGGCAGAAGCA
PaIPT3	CTTTATCCTCGGCTCCACCG	TCGGAGTTGATGACTTCGCC
PaIPT4	AGGTACTGTGGCTGCTTACG	CTACTACAGGCGATGCGTCC
PaIPT5	TCAGATCCTCGGGGTTTCCT	CCTCGCACCGCAGATCAATA
PaIPT6	TGAGACGGTGAGGAGGTTTC	GTTCTCTTCGCTGATCTCCAC
PaLOG1	GTGCCCATGAGTTGCTGTCT	GCTGCTCCATCTCCCAACTC
PaLOG2	TCCTGGTGGGTATGGGACAA	TGCCTTGCTGAGCTCTCTATG
PaLOG3	TGGGCTCAATTAGGCATCCA	GATATGCCGAGCACTTGGACT
PaLOG4	GGTGGCGAGGAACATTGACT	CCGCCATTAAAGACTGCCTG
PaLOG5	GTTGGGGAAGTTCTAGCGGT	TTCCATAGCCACCAGGCAAA
PaLOG6	ATTTGGGCCGAGAACTGGTG	CCAAGAACATCACAACCCCC
PaCKX1	CTGGAGGAAATGGCGATAGG	GGATCAAACCTCCTCTTCAGAT
PaCKX2	CCACGTTCTTCAGGTCAGGA	GGATGGCCAGGGGATCAAAA
PaCKX3	GGCCAACACAACTATGGGTC	CATCTGGAATTGTTGTTGACATCCT
PaCKX4	CCCCTTCCAATCCCGTGAACA	CCGTTGTGAAGACCGAACCC
PaCKX5	GAGGTGGTGACTGGAAAGGG	TGCTGTGGAGCATCTTGGAG
PaCKX6	AAAGCTAACGAGCGGGCCTA	GGGCCGTAACGAAAGGTCTG
PaUbiquitin	TGAACTCCATCGCCTTCCTCTTC	TGAAGCATGGCATCAATTTC

Table S2. *IPT* genes identified from *P. aphrodite* genome, along with their molecular details and relevant genomic information.

Gene name[a]	Locus[b]	Chr[c]	Length[d]	IPPT[e]	IPT[e]	Family[f]	E value[g]	Identity (%)[h]	TargetP[i]	Glycosylation Site[j]
PaIPT1	PAXXG118600	L10a	335	5.37e-31	1.89e-03	AtIPT1	1.89e-94	48.065	MT	0
PaIPT2	PAXXG226240	L08a	502	2.98e-59	-	AtIPT2	3.17e-156	51.982	Other	1
PaIPT3	PAXXG097640	L04	306	1.51e-34	7.26e-03	AtIPT3	1.52e-89	47.97	Other	1
PaIPT4	PAXXG213053	Sca234	284	2.44e-29	1.61e-03	AtIPT8	3.99e-91	46.181	Other	0
PaIPT5	PAXXG302430	L13	449	3.07e-78	1.13e-06	AtIPT9	2.3e-165	53.125	CH	3
PaIPT6	PAXXG097630-	L04	340	8.93e-35	1.34e-04	AtIPT3	2.95e-90	46.392	Other	2

[a]Gene Names given to *PaIPTs* in this work.

[b]Locus represented by the *P. aphrodite* genome database.

[c]Chromosomal localization of the *PaIPTs,* Sca represents scaffold.

[d]Length the number of amino acids

[e]PFAM e-values for having the indicated protein domains. PFAM e-values for having the indicated protein domains. IPT domain means Isopentenyl transferase, and IPPT domain means IPPT transferases in PFAM database.

[f]Closest homologs from *Arabidopsis thaliana*.

[g]Blastp e-values for having the indicated protein domains.

[h]Percentage of identical amino acids with the closest *Arabidopsis thaliana* homologs

[i]Localization predicted with TargetP-2.0. "SP" for signal peptide, "MT" for mitochondrial transit peptide (mTP),"CH" for chloroplast transit peptide (cTP), "TH" for thylakoidal lumen composite transit peptide (lTP),"Other" for no targeting peptide (in this case, the length is given as 0).

[j]Glycosylation sites are predicted with NetNGlyc - 1.0

Table S3. *LOG* genes identified from *P. aphrodite* genome, along with their molecular details and relevant genomic information.

Gene name[a]	Locus[b]	Chr[c]	Length[d]	Lysine_decarbox[e]	Family[f]	E value[g]	Identity (%)[h]	TargetP[i]	Glycosylation Site[j]
PaLOG1	PAXXG136830	L03	221	9.17e-60	AtLOG1	3.81e-122	76.555	Other	1
PaLOG2	PAXXG166050	L05a	244	2.58e-56	AtLOG1	5.12e-110	67.805	Other	0
PaLOG3	PAXXG233850	Sca192	215	1.19e-65	AtLOG1	1.19e-138	85.849	Other	0
PaLOG4	PAXXG280860	Sca468	192	1.20e-58	AtLOG1	4.41e-114	83.240	Other	0
PaLOG5	PAXXG038340	L15	229	3.81e-61	AtLOG7	1.46e-117	80.612	Other	0
PaLOG6	PAXXG106310	L06	225	4.65e-57	AtLOG8	8.9e-110	71.981	Other	0

[a]Gene Names given to *PaLOGs* in this work.
[b]Locus represented by the *P. aphrodite* genome database.
[c]Chromosomal localization of the *PaLOGs*, Sca represents scaffold.
[d]Length the number of amino acids
[e]PFAM e-values for having the indicated protein domains.
[f]Closest homologs from *Arabidopsis thaliana*.
[g]Blastp e-values for having the indicated protein domains.
[h]Percentage of identical amino acids with the closest *Arabidopsis thaliana* homologs
[i]Localization predicted with TargetP-2.0. "SP" for signal peptide,"MT" for mitochondrial transit peptide (mTP),"CH" for chloroplast transit peptide (cTP),"TH" for thylakoidal lumen composite transit peptide (lTP),"Other" for no targeting peptide (in this case, the length is given as 0).
[j]Glycosylation sites are predicted with NetNGlyc - 1.0

Table S4. *CKX* genes identified from *P. aphrodite* genome, along with their molecular details and relevant genomic information.

**Gene name**[a]	**Locus**[b]	**Chr**[c]	**Length**[d]	**FAD_ binding_4**[e]	**CK-binding**[e]	**Family**[f]	**E value**[g]	**Identity (%)**[h]	**TargetP**[i]	**Glycosylation Site**[j]
PaCKX1	PAXXG012950	L15	496	5.11e-23	4.64e-102	AtCKX1	4.14e-134	41.955	SP	2
PaCKX2	PAXXG031040	L10a	520	5.47e-19	8.73e-172	AtCKX1	0	64.155	SP	4
PaCKX3	PAXXG242550	Sca318	529	4.64e-23	6.56e-163	AtCKX3	1.58e-176	52.119	SP	0
PaCKX4	PAXXG031510	L10a	527	2.01e-17	0e+00	AtCKX5	0	69.535	SP	3
PaCKX5	PAXXG232040	Sca286	544	3.99e-19	5.85e-143	AtCKX6	6.51e-152	46.951	SP	0
PaCKX6	PAXXG098230	L01	509	1.47e-31	2.62e-145	AtCKX7	0	55.907	Other	2

[a]Gene Names given to *PaCKXs* in this work.
[b]Locus represented by the *P. aphrodite* genome database.
[c]Chromosomal localization of the *PaCKXs*, Sca represents scaffold.
[d]Length the number of amino acids.
[e]PFAM e-values for having the indicated protein domains.
[f]Closest homologs from *Arabidopsis thaliana*.
[g]Blastp e-values for having the indicated protein domains.
[h]Percentage of identical amino acids with the closest *Arabidopsis thaliana* homologs
[i]Localization predicted with TargetP-2.0. "SP" for signal peptide,"MT" for mitochondrial transit peptide (mTP),"CH" for chloroplast transit peptide (cTP),"TH" for thylakoidal lumen composite transit peptide (lTP),"Other" for no targeting peptide (in this case, the length is given as 0).
[j]Glycosylation sites are predicted with NetNGlyc - 1.0

Figure S1. Western blot assay showing the production of PaCKX4, PaLOG2, PaIPT1, and green fluorescent protein (GFP, the control) in the agroinfiltrated *N. benthamiana* leaves.

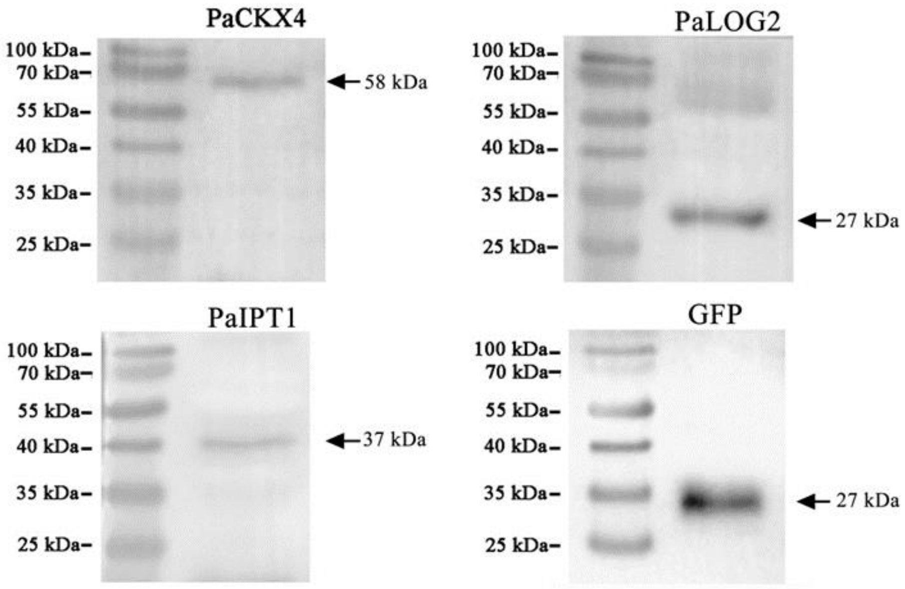

Supplement: Supplementary file 1 [file ijms-23-03755-s001.zip › ijms-1532904-supplementary.pdf]
